# Monkeypox virus protein OPG188 antagonizes cGAS–STING antiviral signaling pathway to mediate immune evasion

**DOI:** 10.1073/pnas.2523334123

**Published:** 2026-03-18

**Authors:** Zhaoyi Pan, Shujuan Zhang, Xianbo Geng, Na Wang, Lijiang Zhang, Luyao Wang, Chunhong Yin, Huijiao Zhang, Shujun Liu, Ling Zhang, Jing Fan, Guangjian Xue, Rui Li, Tianle Li, Yating Yu, Hangping Yao, Changzhong Jin, Nanping Wu

**Affiliations:** ^a^Jinan Microecological Biomedicine Shandong Laboratory, Jinan 250118, China; ^b^Zhejiang Key Laboratory of High-level Biosafety and Biomedical Transformation, Hangzhou Medical College, Hangzhou 311305, China; ^c^Infectious Disease Control Institute, Shandong Center for Disease Control and Prevention, Jinan 250014, China; ^d^Shandong Provincial Key Laboratory of Intelligent Monitoring, Early Warning, Prevention and Control for Infectious Diseases, Jinan 250014, China; ^e^Shandong First Medical University and Shandong Academy of Medical Sciences, Jinan 250117, China; ^f^State Key Laboratory for Diagnosis and Treatment of Infectious Diseases, National Clinical Research Center for Infectious Diseases, Collaborative Innovation Center for Diagnosis and Treatment of Infectious Diseases, The First Affiliated Hospital, Zhejiang University School of Medicine, Hangzhou 310006, China

**Keywords:** monkeypox virus, cGAS–STING, OPG188, Theaflavin-3’-gallate, NAD^+^

## Abstract

Innate immune evasion is critical for productive viral replication. Activation of the cGAS–STING antiviral signaling pathway and its downstream effector genes plays a pivotal role in restricting viral replication during early DNA virus infection. Through a comprehensive genomic screen of monkeypox virus (MPXV), we identified three viral genes, OPG147, OPG188, and OPG200, whose expression potently suppresses cGAS–STING pathway activation. Notably, the N-terminal domain of the Poxin protein encoded by OPG188 exhibits nuclease activity and cleaves the cyclic dinucleotide second messenger 2′3′-cGAMP. Using site-directed mutagenesis, we further delineated nine conserved regions and four key amino acid residues—H15, K140, R182, and I79—within Poxin that are essential for antagonism of cGAS–STING signaling. Moreover, via molecular docking and high-throughput screening of 7,155 small molecules targeting the catalytic pocket of Poxin, we identified two compounds that competitively bind to Poxin, inhibiting its cGAMP—degrading activity and consequently restoring cGAS–STING-mediated antiviral signaling upon MPXV infection. Collectively, these findings underscore the pivotal role of OPG188 in modulating antiviral immune responses and highlight NAD^+^ and Theaflavin-3’-gallate as promising candidates for the development of anti-MPXV therapeutics.

## Significance

This study identifies key monkeypox virus (MPXV) immune evasion proteins—OPG147, OPG188, and OPG200—that suppress the host cGAS–STING antiviral pathway. We characterize OPG188 as a nuclease that degrades the immune signaling molecule 2′3′-cGAMP and define critical functional residues via mutagenesis. Using structure-based screening, we find two small molecules (NAD^+^, Theaflavin-3’-gallate) that block Poxin activity, restoring antiviral signaling and highlighting their potential as therapeutics against MPXV infection. These findings provide crucial insights into poxvirus immune evasion and open avenues for developing virus-directed antiviral strategies.

Historically, monkeypox virus (MPXV) has been a zoonosis with little human-to-human transmission and until 2022 was largely limited to regions of Africa. Between July 2022 and May 2023, more than 99,000 confirmed cases from 115 countries around the world were reported (1). On August 14, 2024, the World Health Organization declared MPXV a Public Health Emergency of International Concern for a second time due to a rapid increase in infections with clade I MPXV in Central Africa (https://www.who.int/zh/news/item/14-08-2024-who-director-general-declares-mpox-outbreak-a-public-health-emergency-of-international-concern). MPXV is a large double-stranded DNA virus that belongs to the *Orthopoxvirus* genus of the Poxviridae family, which also includes variola virus, vaccinia virus (VACV), cowpox virus (CPXV) (2, 3).

Pattern recognition receptors (PRRs) serve as the first line of host defense by initiating innate immune responses against invading pathogens. Upon cellular entry, MPXV undergoes DNA replication exclusively within the cytoplasm of infected cells (4). A key cytosolic DNA sensor, cyclic GMP-AMP synthase (cGAS), utilizes ATP and GTP to synthesize the second messenger 2′3′-cGAMP—a potent agonist of the stimulator of interferon genes (STING). Binding of 2′3′-cGAMP to ER-localized STING triggers its translocation from the ER membrane through the Golgi apparatus to post-Golgi compartments. At these sites, STING recruits and activates the kinase TBK1, which in turn phosphorylates the transcription factor IRF3 (5–7). This modification promotes IRF3 dimerization and nuclear translocation, leading to the transcriptional induction of type I interferon genes (8). Nevertheless, the mechanisms through which MPXV evades the cGAS–STING-mediated antiviral immune pathway remain poorly understood.

Poxin homologs widely exist in the genome of invertebrate DNA and RNA viruses, as well as in hymenopteran parasitoid wasps and lepidopteran insects (9, 10). In 2019, the poxin protein encoded by the vaccinia virus gene B2R was identified as a 2′3′-cGAMP-specific nuclease that potently suppresses the cGAS–STING antiviral signaling pathway (9). Subsequent evolutionary analyses indicate that although many poxin-like proteins retain the ability to degrade 2′3′-cGAMP, a significant subset has lost this activity. OPG188 is a close homolog of known poxin immune evasion proteins from orthopoxviruses. Nevertheless, the functional characterization of OPG188 from MPXV remains incomplete, primarily due to difficulties in achieving recombinant expression in *Escherichia coli* (11). In orthopoxviruses, poxin is expressed as an early gene, either as a standalone protein or as a C-terminal fusion with a viral schlafen protein—a known inhibitor of STING (12). A recent study further revealed that the MPXV poxin-schlafen fusion protein counteracts host antiviral responses by sequestering STAT2, rather than by suppressing cGAS or 2′3′-cGAMP-mediated induction of type I interferons (IFN-I) (13).

In this study, we conducted a comprehensive screen to identify MPXV genes capable of modulating the cGAS–STING axis and identified OPG147, OPG188, and OPG200 as three potent inhibitors. Among these, OPG188 encodes a poxin-schlafen fusion protein. Overexpression of OPG188 specifically suppressed innate antiviral responses triggered by viral DNA, but not those induced by RNA. Conversely, knockdown of OPG188 led to enhanced induction of IFN-I and downstream antiviral genes. Furthermore, we identified nine conserved regions and four critical amino acid residues-H15, K140, R182, and I79-within OPG188 that are essential for its antagonistic effect on cGAS–STING pathway activation. Finally, we found two compounds that significantly inhibit OPG188 activity through competitive binding to its catalytic active sites.

## Materials and Methods

### Cells and Viruses.

All cell lines and viruses, along with the corresponding culture conditions and sources, are detailed in *SI Appendix*.

### Reagents and Antibodies.

Detailed information on the sources and catalog numbers of all reagents and antibodies used is provided in the *SI Appendix*.

### Constructs.

All plasmid sources and construction details are provided in the *SI Appendix*.

### Constructs of Stable Cell Lines.

The procedures for generating knockdown and overexpression cell lines are detailed in the *SI Appendix*.

### Transfection and Reporter Assays.

Detailed procedures for the transfection and reporter assays are provided in the *SI Appendix*.

### Screening for OPG188 Antagonist Compounds By Luciferase Assay.

HEK293T cells (1 × 10^5^) were transfected with an IFN-β reporter (50 ng) + pRL-TK (5 ng) along with cGAS (50 ng) + STING (50 ng) or an empty vector, and OPG188 plasmids (50 ng). Compounds (final concentration: 100 μM) were added 6 h posttransfection, followed by 18 h of incubation before luciferase assay.

### qPCR.

Total RNA was extracted using TRIzol reagent and reverse-transcribed to cDNA for qPCR analysis to measure mRNA levels of the indicated genes. The mRNA levels of the tested genes were normalized to *18S* rRNA levels. Gene-specific primer sequences are detailed in the *SI Appendix*.

### Immunoblot Analysis.

HEK293T or THP-1 cells were lysed in SDS lysis buffer. The lysates were incubated at 97 °C for 15 min and analyzed using standard immunoblot procedures as described previously (14).

### SDD-AGE Assay.

The cells were washed with PBS and resuspended in lysis buffer. The supernatants were separated by 1.5% SDD-AGE. After electrophoresis in the running buffer (1 × TBE and 0.1% SDS) for 60 minutes with a constant voltage of 100 V at 4 °C, the proteins were transferred to 0.45 μm PVDF membrane (Millipore, ZY101123) for immunoblotting.

### Coimmunoprecipitation.

HEK293T cells (~10^7^) were lysed with 1 ml NP-40 lysis buffer for 30 minutes on ice. The lysates were clarified by centrifugation at 12,000 rpm for 10 min at 4 °C and subsequently used for coimmunoprecipitation as detailed in *SI Appendix*.

### Confocal microscopy.

HEK293T cells were transfected with either OPG188 or an empty vector, in conjunction with cGAS, STING, and mCherry-GalT, for 24 h. The cells were then processed and observed by confocal microscopy, as detailed in the *SI Appendix*.

### Detection of 2’3’-cGAMP by Mass Spectrometry and ELISA.

Detailed procedures for the detection of 2′3′-cGAMP using mass spectrometry and ELISA are provided in the *SI Appendix*.

### OPG188 Protein Purification.

HEK293T cells (around 1 × 10^6^ cells per dish) cultured in 10-cm dishes were transfected with 10 µg of pCDNA3.1-OPG188-6 × His plasmid. After 36 h, cells from ten 10 cm dishes were harvested and lysed using NP-40 cell lysis buffer supplemented with protease inhibitor cocktail. The lysates were subsequently used for purification of the OPG188 protein. Detailed purification procedures are provided in *SI Appendix*.

### Docking and Virtual Screening.

Protocols for Docking and Virtual Screening can be found in *SI Appendix*.

### NMN or TF2B Against OPG188 Nuclease Activity Assays In Vitro.

Purified OPG188 (200 ng) was incubated with varying concentrations of NAD^+^/TF2B for 10 minutes at 37 °C in a 50 μL reaction buffer containing 50 mM HEPES-KOH (pH 7.5), 35 mM KCl, and 1 mM DTT. Detailed experimental procedures are provided in the *SI Appendix*.

### MPXV Infection of Mice.

All animal experiments were conducted using BALB/c mice (female, 6 wk) housed in an Animal Biosafety Level 3 facility. Details regarding experimental groups, treatments, and other procedures are provided in the *SI Appendix*.

### Ethics Statement.

All animal experiments were approved by and performed in accordance with the guidelines of Hangzhou Medical College Animal Care and Use Committee. All experiments with live MPXV were performed in the BSL-3 facilities of Hangzhou Medical College Animal Experiment Center (approval number: 202502053).

### Statistical Analysis.

Statistical significance was calculated using an unpaired Student’s *t* test (two-tailed), *P < 0.05, **P < 0.01, ***P < 0.001, ****P < 0.001. Results are shown as arithmetic means ± SD. GraphPad Prism 9 was used for statistical analysis.

## Results

### OPG147, OPG188, and OPG200 Inhibit cGAS-Mediated Signaling.

**Fig. 1.**
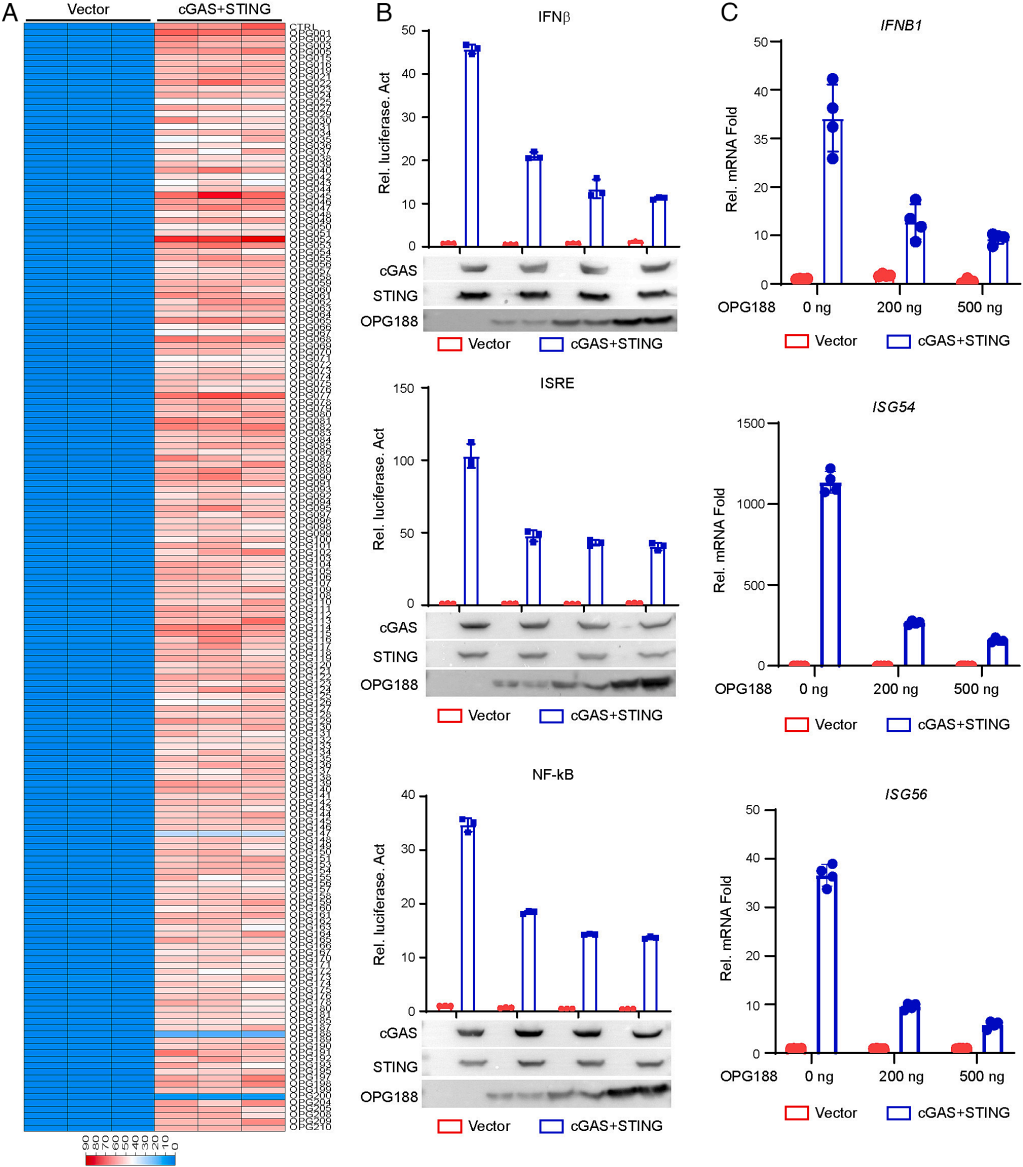
Identification of MPXV OPG188 as an Inhibitor of cGAS–STING-Mediated Signaling. (*A*) Screening for MPXV-encoded proteins that modulate cGAS–STING signaling. HEK293T cells (1 × 10^5^) were cotransfected with expression plasmids for cGAS, STING, an IFN-β luciferase reporter (0.05 µg), the pRL-TK control reporter (0.005 µg), and the indicated MPXV protein-expressing constructs (0.05 µg). After 24 h, cells were harvested and luciferase activity was measured. (*B*) OPG188 suppresses cGAS–STING-mediated activation of the *IFNB* promoter, ISRE, and NF-κB in a dose-dependent manner. HEK293T cells (1 × 10^5^) were transfected with the indicated reporter constructs, along with cGAS and STING or an empty vector, together with increasing amounts of OPG188 plasmid. Luciferase assays were performed 24 h posttransfection. Protein expression levels were verified by immunoblotting. (*C*) OPG188 inhibits cGAS–STING-induced transcription of antiviral genes in a dose-dependent manner. HEK293T cells were transfected with cGAS and STING or an empty vector, together with increasing amounts of OPG188 plasmid. After 24 h, mRNA levels of antiviral genes were analyzed by quantitative PCR. ns, not significant; *P < 0.05 and **P < 0.01; Student’s *t* test, n ≥3

The cGAS–STING pathway is widely recognized as a central signaling axis in the innate immune response to DNA virus infection (15–18). To identify MPXV-encoded proteins that modulate cGAS-dependent signaling, we constructed a library of expression clones encoding each of the 179 MPXV genes and screened them for the ability to suppress cGAS–STING-driven activation of the IFNB promoter in HEK293T cells. This screen identified OPG147, OPG188, and OPG200 as potential inhibitors (Fig. 1*A*). It has been previously shown that OPG147 as a virulence factor inhibits cGAS–STING activation (19). Besides, no studies to date have reported on the regulation of the cGAS–STING pathway by OPG200. However, there is an ongoing debate regarding the potential role of MPXV OPG188 in regulating the cGAS–STING antiviral pathway (13, 19). Therefore, we first focused our investigation on OPG188 in this study. Further experiments showed that OPG188 inhibited cGAS–STING-mediated activation of IFNβ/interferon-stimulated response element (ISRE, which is bound by activated IRF3) /NF-κB in a dose-dependent manner (Fig. 1*B*). Consistently, qPCR experiments indicated that overexpression of OPG188 inhibited cGAS–STING induced transcription of *IFNB1*, *ISG54*, and *ISG56* genes (Fig. 1*C*). These data suggest that MPXV-OPG188 is an inhibitor of cGAS–STING mediated signaling.

### OPG188 Inhibits DNA Virus–Triggered Induction of Downstream Antiviral Genes.

**Fig. 2.**
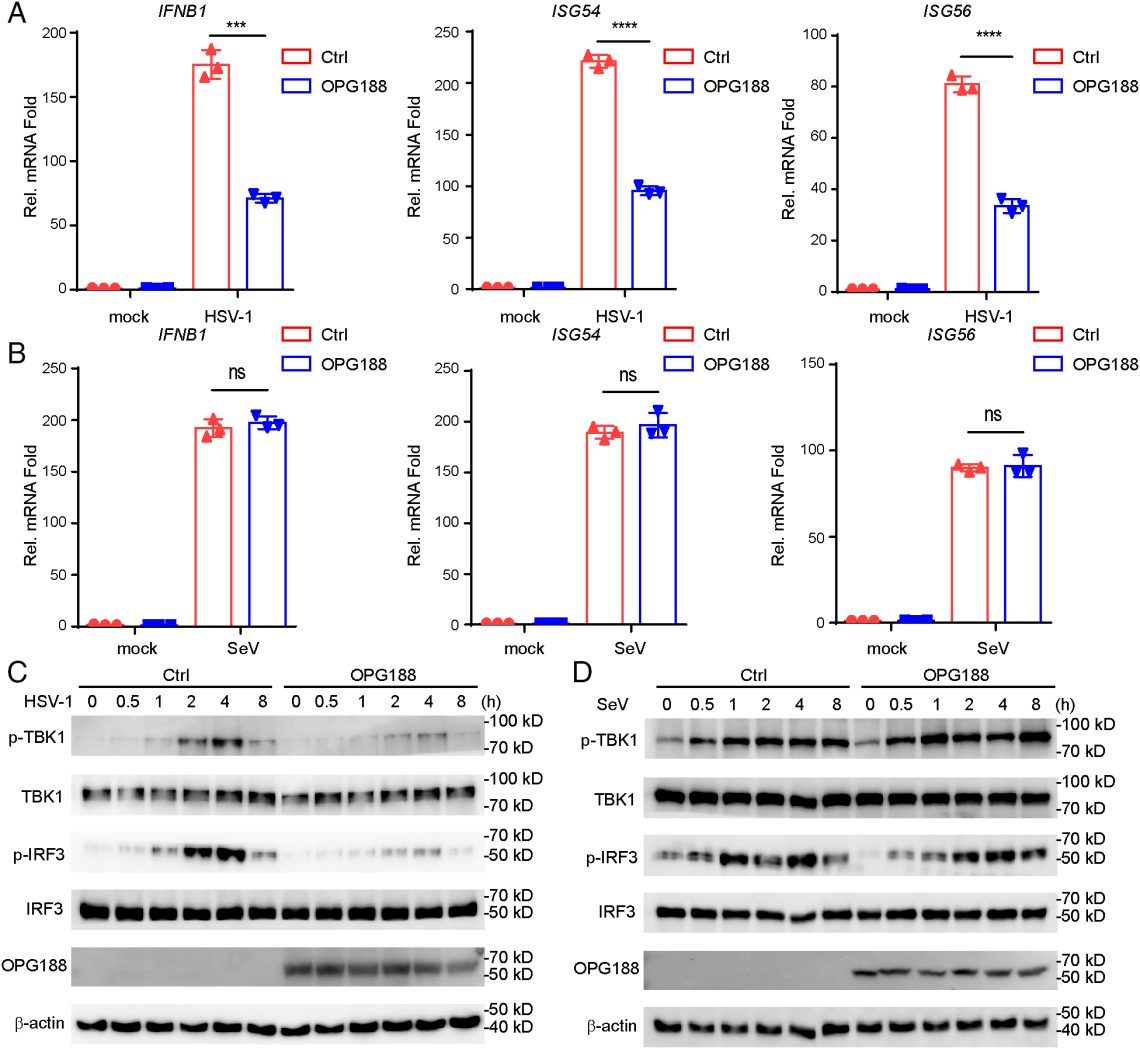
OPG188 Negatively Regulates Viral DNA-Induced Antiviral Response. (*A* and *B*) OPG188 suppresses HSV-1- but not SeV-triggered expression of antiviral genes. THP-1 cells stably expressing OPG188-Flag or an empty vector were infected with HSV-1 (*A*) or SeV (*B*) (MOI = 1) for the indicated durations and analyzed by qPCR. Data are presented as mean ± SD; ns, not significant; *P < 0.05, **P < 0.01 (Student’s *t* test; n = 3). (*C* and *D*) OPG188 inhibits HSV-1- but not SeV-induced phosphorylation of TBK1 and IRF3. THP-1 cells stably expressing OPG188-Flag or a control vector were infected with HSV-1(*C*) or SeV (*D*) (MOI = 1) for the indicated times. Immunoblotting was conducted using anti-Flag and antibodies specific to the indicated proteins.

To further investigate the role of viral OPG188 in innate antiviral response, we generated a stable THP-1 cell line expressing OPG188. It has been previously reported that HSV-1 infection triggered cGAS–STING antiviral signaling pathway in THP-1 cell (20–23). Following HSV-1 infection, THP-1 cell line stably expressing OPG188 showed impaired transcription of *IFNB1*, *ISG54*, and *ISG56* genes compared with empty vector-transduced control cells (Fig. 2*A*). However, in similar experiments, transcriptions of *IFNB1*, *ISG54*, and *ISG56* genes induced by Sendai Virus (SeV), which is an RNA virus, showed no significant difference between OPG188-expressing cells and control cells (Fig. 2*B*). As the important adapter downstream of the cGAS–STING antiviral signaling pathway, TBK1 and IRF3 phosphorylation are hallmarks of the activation of cGAS-mediated signaling. Consistently, OPG188 inhibited phosphorylation of TBK1 and IRF3 in response to HSV-1 but not SeV infection (Fig. 2 *C* and *D*). These results indicate that OPG188 specifically inhibits the innate immune signaling pathway triggered by DNA virus, but not by RNA virus.

### OPG188-Knockdown Potentiates cGAS–STING Antiviral Signaling to MPXV.

**Fig. 3.**
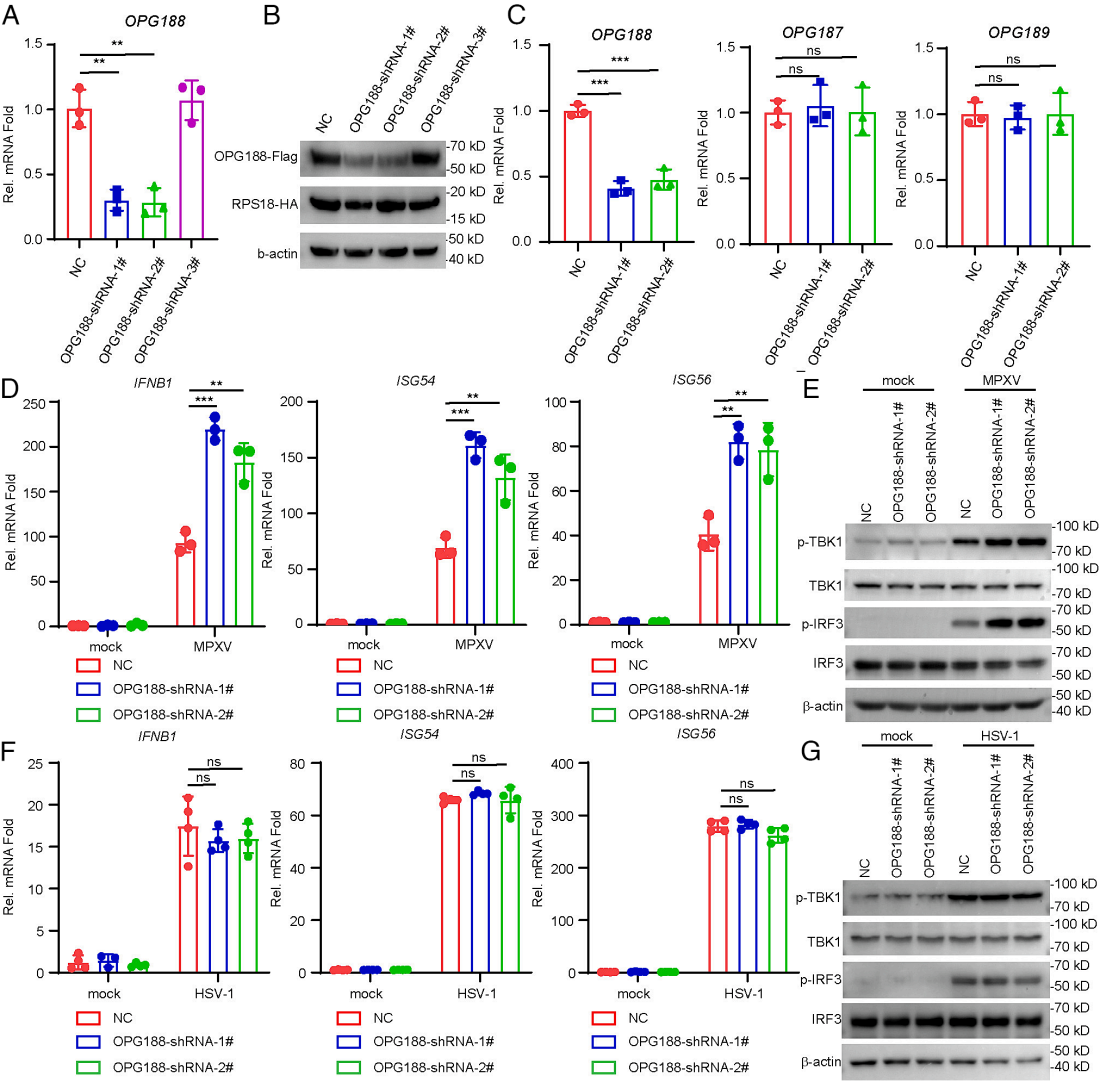
OPG188-knockdown potentiates cGAS–STING antiviral signaling to MPXV. (*A*–*C*) Specific knockdown of OPG188 expression by OPG188-shRNA. (*A* and *B*) HEK293T cells were cotransduced with OPG188-Flag and RPS18-HA, along with either the indicated OPG188-shRNA or a control shRNA, for 36 h before analysis by qPCR (*A*) and immunoblotting (*B*). (*C*) Vero cells stably expressing either the indicated OPG188-shRNA or a control shRNA were infected with MPXV (MOI = 1) for 24 h and then analyzed by qPCR. Data are presented as mean ± SD; n = 3. ns, not significant; *P < 0.05, **P < 0.01 (Student’s *t* test). (*D*–*G*) Knockdown of OPG188 enhances MPXV but not HSV-1-induced transcription of antiviral genes and phosphorylation of TBK1 and IRF3. THP-1 cells stably expressing either the indicated OPG188-shRNA or a control shRNA were either infected with MPXV (MOI = 1) (*D* and *E*), HSV-1 (MOI = 1) (*F* and *G*) or left uninfected for 24 h before analysis by qPCR (*D* and *F*) and immunoblotting (*E* and *G*).

To investigate the role of endogenous OPG188 in the innate immune response to MPXV, we constructed three independent small hairpin RNA (shRNA) plasmids targeting OPG188. qPCR and western blot analysis confirmed that shRNA constructs #1 and #2, but not #3, significantly reduced OPG188 expression (Fig. 3 *A* and *B*). Therefore, we generated Vero and THP-1 cell lines stably expressing either #1 or #2 OPG188-targeting shRNA, along with a control shRNA. In Vero cells, which are deficient in IFN-I signaling, both OPG188 shRNAs specifically knocked down endogenous OPG188 mRNA without affecting the expression of the adjacent genes OPG187 and OPG189 (Fig. 3*C*). MPXV infection of OPG188-knockdown THP-1 cells resulted in markedly enhanced transcription of *IFNB1*, *ISG54*, and *ISG56* at 24 h postinfection compared to control cells (Fig. 3*D*). Consistent with these findings, OPG188 knockdown also promoted MPXV-induced phosphorylation of TBK1 and IRF3 in THP-1 cells (Fig. 3*E*). However, in similar experiments, transcriptions of *IFNB1*, *ISG54*, *ISG56* genes and phosphorylation of TBK1, IRF3 induced by HSV-1 showed no significant difference between control and OPG188-knockdown THP-1 (Fig. 3). These results suggest that MPXV-OPG188 negatively regulates innate immune response to MPXV infection in THP-1 cells.

One recent study has shown that MPXV-OPG188 suppresses IFN-I stimulated activity of interferon-stimulated response elements but not cGAS–STING-mediated IFN-I production by sequestering STAT2 (13). Consistent with these findings, our data indicate that OPG188 interacts with STAT2, yet does not attenuate IFNβ-induced transcription of *ISG15*, *ISG54*, or *ISG56* (*SI Appendix*, Fig. S1 *A* and *B*). Laser scanning confocal microscopy further revealed that OPG188 primarily localizes in aggregated forms in resting cells, but redistributes diffusely throughout the cytoplasm upon activation of the STING pathway (*SI Appendix*, Fig. S1*C*). Upon activation of the STING signaling pathway, the observed repositioning of OPG188 may be inconsistent with its previously reported colocalization with STAT2 (13).

### OPG188 Disrupts 2′3′-cGAMP to Inhibit cGAS–STING Antiviral Signaling.

**Fig. 4.**
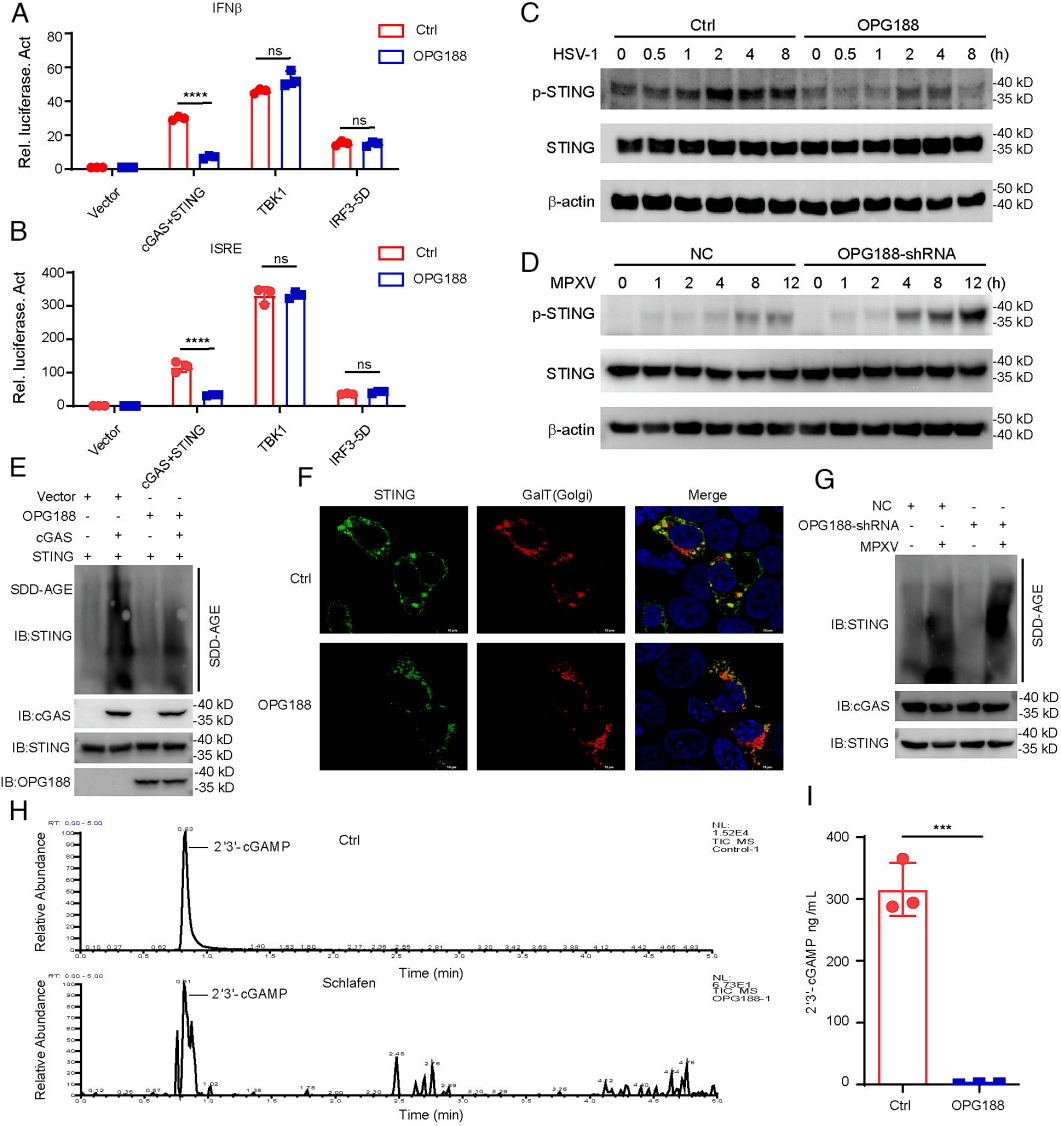
OPG188 disrupts 2′3′-cGAMP to inhibit cGAS–STING antiviral signaling. (*A* and *B*) Activation of the IFNB promoter (*A*) and ISRE (*B*) by cGAS–STING, TBK1, and IRF3-5D in HEK293T cells. (*C*) OPG188 inhibits HSV-1-induced phosphorylation of STING. THP-1 cells stably expressing OPG188-Flag or an empty vector control were infected with HSV-1 (MOI = 1) for the indicated durations. Immunoblotting was performed using antibodies against the indicated proteins. (*D*) Knockdown of OPG188 enhances MPXV-induced phosphorylation of STING. THP-1 cells stably expressing either control shRNA or OPG188-targeting shRNA were infected with MPXV (MOI = 1) for the indicated times. Immunoblotting was conducted with the indicated antibodies. (*E*) HEK293T cells were transfected with the indicated plasmids. After 24 h, cells were analyzed by SDD-AGE and SDS-PAGE followed by immunoblotting with the indicated antibodies. (*F*) OPG188 inhibits STING translocation from the ER to the Golgi apparatus. HEK293T cells were cotransfected with cGAS and STING along with either OPG188 or an empty vector for 24 h and then examined by confocal microscopy. (*G*) THP-1 cells stably expressing control shRNA or OPG188-targeting shRNA were infected with MPXV (MOI = 1) for 12 h and subsequently analyzed by SDD-AGE and SDS-PAGE followed by immunoblotting with the indicated antibodies. (*H*) Measurement of 2′3′-cGAMP levels via mass spectrometry. (*I*) Quantitative analysis of 2′3′-cGAMP degradation by OPG188.

To elucidate the molecular mechanism by which MPXV-OPG188 impairs IFN-I production, we evaluated the effect of OPG188 on the activation of the IFNβ and ISRE promoter in HEK293T cells stimulated by cGAS+STING, TBK1 or IRF3-5D (a constitutively active form of IRF3) (24, 25) and *IFNB1*, *ISG54*, *ISG56* expression in THP-1 stimulated by poly(dA:dT) or 2′3′-cGAMP. As shown in Fig. 4 *A* and *B* and *SI Appendix*, Fig. S2*A*, ectopic expression of OPG188 suppressed both IFNβ promoter, ISRE activity, or *IFNB1*, *ISG54*, *ISG56* expression triggered by poly(dA:dT), cGAS–STING, 2′3′-cGAMP, but not by TBK1 or IRF3-5D, suggesting that OPG188 acts upstream of TBK1 in the cGAS–STING signaling cascade. Consistently, phosphorylation of STING was inhibited upon HSV-1 infection in THP-1 cells expressing OPG188, whereas STING phosphorylation was enhanced in OPG188-knockdown THP-1 cells infected with MPXV (Fig. 4 *C* and *D*). Given that binding of 2′3′-cGAMP to STING promotes its aggregation and Golgi translocation (5, 6, 26, 27), we examined whether OPG188 affects these processes. Overexpression of OPG188 markedly suppressed STING aggregation and Golgi localization (Fig. 4 *E* and *F*), whereas OPG188 knockdown enhanced STING aggregation (Fig. 4*G*), implying that OPG188 may interfere with the interaction between STING and 2′3′-cGAMP. Previous studies have revealed that most poxviruses encode a Poxin protein with the potential to degrade 2′3′-cGAMP (9). Since OPG188 also contains a Poxin domain, we further investigate whether OPG188 functions as a 2′3′-cGAMP-specific degrading enzyme, we performed an in vitro degradation assay. The results demonstrated that OPG188 significantly degrades 2′3′-cGAMP (Fig. 4 *H* and *I*). Furthermore, knockdown of OPG188 expression enhanced intracellular 2′3′-cGAMP levels (*SI Appendix*, Fig. S2*B*).

### OPG188 H15/ K140/R182 Is Essential for Antagonizing cGAS–STING Signaling.

**Fig. 5.**
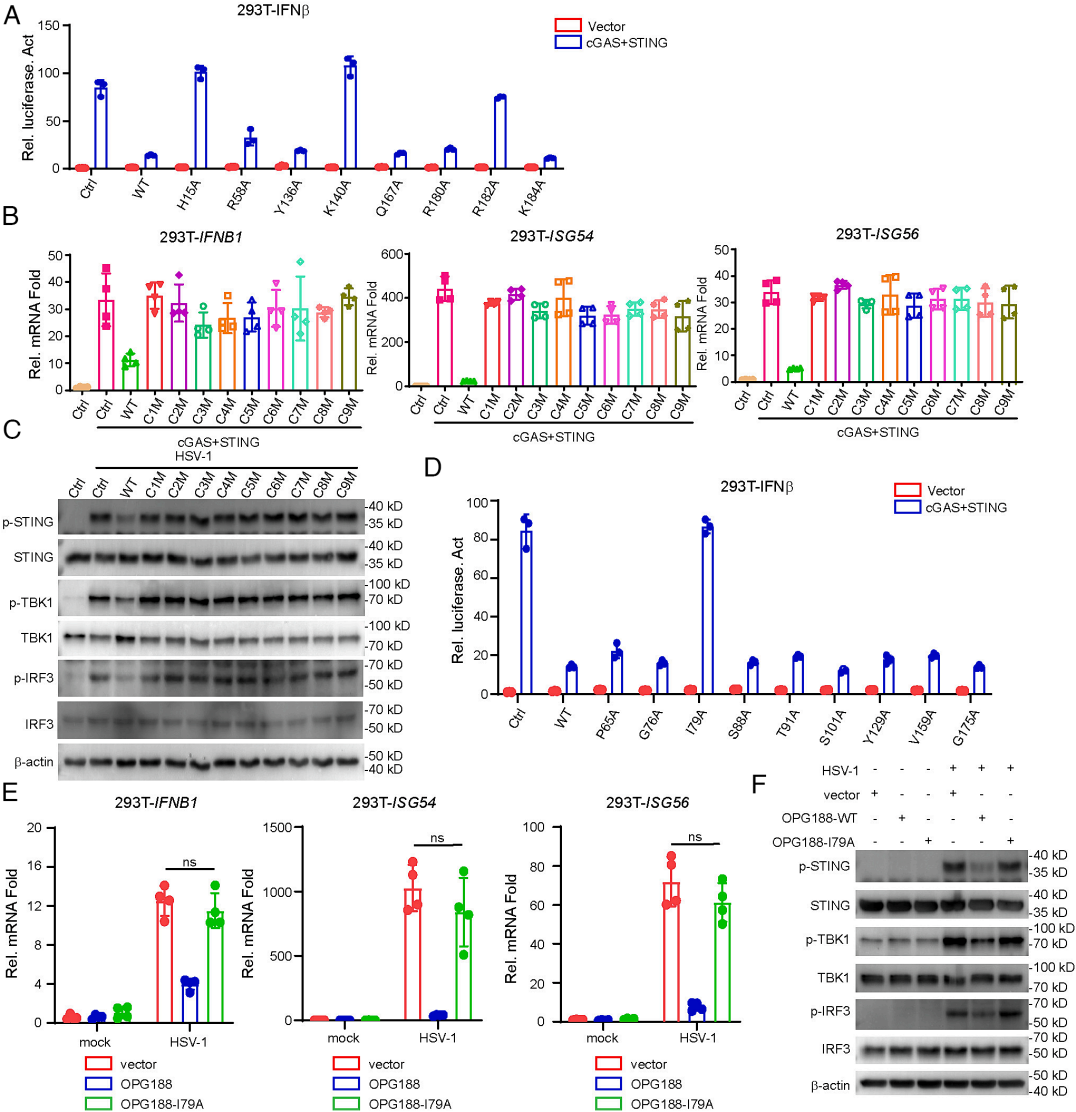
Conserved region and amino acid residues essential for OPG188 disrupting 2′3′-cGAMP. (*A*) HEK293T cells (1 × 10^5^) were transfected with an IFN-β reporter, cGAS + STING or an empty vector, and either OPG188-WT or site-directed mutant plasmids. Luciferase assays were conducted after 24 h. (*B*) HEK293T cells were cotransfected with either OPG188-WT, a conserved region mutant, or an empty vector, along with cGAS+STING or an empty vector, for 24 h prior to qPCR analysis. (*C*) THP-1 cells stably expressing OPG188-WT, a conserved region mutant, or an empty vector were infected with HSV-1 (MOI = 1) for 12 h. Immunoblotting was performed using antibodies against the indicated targets. (*D*) HEK293T cells (1 × 10^5^) were transfected with an IFN-β reporter, cGAS + STING or an empty vector, and either OPG188-WT or site-directed mutant plasmids. Luciferase assays were conducted after 24 h. (*E* and *F*) The I79A mutation impairs the ability of OPG188 to suppress HSV-1-induced antiviral gene expression and phosphorylation of STING, TBK1, and IRF3. THP-1 cells stably expressing OPG188-WT, OPG188-I79A, or a control vector were infected with HSV-1 (MOI = 1) or left uninfected for 12 h, followed by qPCR (*E*) and immunoblotting (*F*) analysis.

Poxin is highly conserved across a wide range of species, including orthopoxviruses, parasitoid wasps, numerous lepidopterans, as well as multiple lepidopteran viruses such as Alphabaculoviruses and Betabaculoviruses. Most Poxin-containing proteins exhibit nuclease activity specifically targeting 2′3′-cGAMP for degradation (9). As a nuclease, we speculate that OPG188 relies on two key aspects for its function. First, it depends on critical amino acid residues in the catalytic active site. Previous studies have established that VACV poxin residues H17, R60, Y138, K142, R182, and R184 are essential for 2′3′-cGAMP degradation (9). By mutating the homologous residues in MPXV-OPG188 (*SI Appendix*, Fig. S3*A*), we found that only H15, K140, and R182 are critical for its antagonistic function against cGAS–STING-mediated activation of the *IFNB* promoter in HEK293T cells (Fig. 5*A*). Second, conserved regions and amino acid residues outside the catalytic site are also crucial for OPG188’s activity by maintaining its proper conformation. Given that OPG188 contains a Poxin domain at its N-terminus, we analyzed the homology of poxin protein sequences from 12 different species. Based on the alignment of conserved amino acid residues, we categorized the evolutionary conservation of Poxin into nine distinct regions, designated C1 to C9 (*SI Appendix*, Fig. S3*B*). We performed site-directed mutagenesis on conserved residues within these regions (*SI Appendix*, Table S1). Mutations at these conserved sites abrogated the ability of OPG188 to antagonize cGAS–STING-induced transcription of *IFNB1*, *ISG54*, and *ISG56* (Fig. 5*B*). Western blot analysis further confirmed that these mutations eliminated the suppressive effect of OPG188 on the activation of the cGAS–STING antiviral signaling pathway (Fig. 5*C*).

### MPXV-OPG188 I79 Is Essential for Antagonizing cGAS–STING Signaling.

Through homology analysis, we also identified several conserved amino acid residues among Poxins. Our results demonstrate that residue I79 in MPXV-OPG188 is critical for antagonizing cGAS–STING-mediated activation of the *IFNB* promoter (Fig. 5*D*). We further generated homologous mutations at the corresponding sites in Poxins from VACV and CPXV (equivalent to I79 in MPXV-OPG188). Interestingly, qPCR assays revealed that neither VACV-Poxin-I81 nor CPXV-Poxin-I81 impaired the antagonistic function against cGAS–STING signaling (*SI Appendix*, Fig. S3 *C* and *D*). To further validate the functional significance of I79 in MPXV-OPG188, we generated stable THP-1 cell lines ectopically expressing the OPG188-I79A mutant via lentiviral transduction. Upon HSV-1 infection, the I79 mutant failed to suppress the transcription of *IFNB1*, *ISG54*, and *ISG56* (Fig. 5*E*). Similarly, its ability to inhibit phosphorylation of STING, TBK1, and IRF3 in response to HSV-1 was abolished (Fig. 5*F*). These findings indicate that the antagonistic role of I79 in modulating cGAS–STING signaling is specific to MPXV among the poxviruses tested.

### NAD^+^ and TF2B attenuate the cGAS–STING Signaling Inhibition Caused By OPG188.

**Fig. 6.**
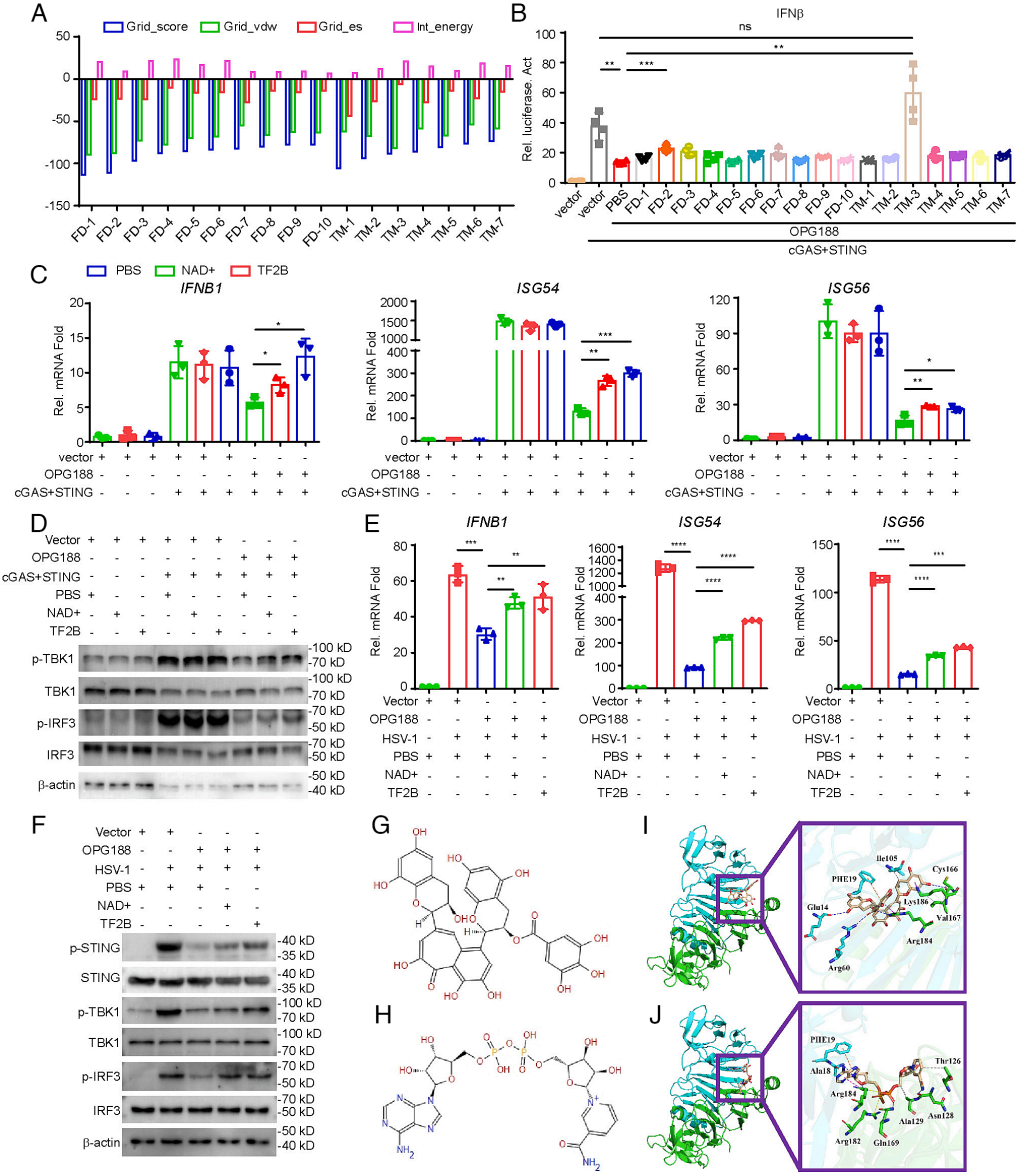
NAD^+^ and Theaflavin-3’-gallate Antagonize OPG188-Mediated Regulation of the cGAS–STING Signaling Pathway. (*A*) Top 17 candidate compounds (FD: FDA library; TM: TargetMol library) identified through molecular docking and virtual screening. Grid_Score: comprehensive score reflecting intermolecular binding affinity; Grid_vdw: van der Waals forces; Grid_es: electrostatic interactions. (*B*) HEK293T cells were transfected with an IFN-β reporter, along with cGAS + STING or an empty vector, and OPG188 plasmids. Compounds were added 6 h posttransfection, followed by 18 h of incubation before luciferase assay. (*C* and *D*) HEK293T cells were transfected with the indicated plasmids. NAD^+^ and Theaflavin-3’-gallate (final concentration: 100 μM) were added 6 h posttransfection, followed by 18 h of incubation prior to qPCR (*C*) and immunoblotting (*D*). (*E* and *F*) THP-1 cells stably expressing OPG188 or a control vector were treated with NAD^+^ and Theaflavin-3’-gallate (final concentration: 100 μM), then infected with HSV-1 (MOI = 1) or left uninfected for 12 h before qPCR (E) and immunoblotting (F) analysis. (*G* and *H*) Chemical structures of Theaflavin-3’-gallate (*G*) and NAD^+^ (*H*). (*I* and *J*) Structural models of Theaflavin-3’-gallate (*I*) and NAD^+^ (*J*) in complex with OPG188.

As a nuclease, OPG188 antagonizes the cGAS–STING signaling pathway through the degradation of 2′3′-cGAMP. Using molecular docking-based virtual screening, we screened the active pocket region of OPG188 against two compound libraries: the FDA library (2,858 compounds) and the TargetMol library (4,279 compounds). Through manual analysis and selection, we identified 17 potential hits (10 from the FDA library and 7 from the TargetMol library) (Fig. 6*A* and *SI Appendix*, Fig. S4). Among these candidates, FD-2 (β-Nicotinamide adenine dinucleotide (NAD^+^), CAS No. 53-84-9) and TM-3 (Theaflavin-3′-gallate (TF2B), CAS No. 28543-07-9) rescued the OPG188-mediated suppression of the *IFNB* promoter (Fig. 6*B*). Furthermore, qPCR assays revealed that both NAD^+^ and TF2B partially reversed the inhibitory effect of OPG188 on the transcription of *IFNB1*, *ISG54*, and *ISG56* (Fig. 6*C*). Consistently, these two compounds also attenuated the OPG188-induced suppression of TBK1 and IRF3 phosphorylation (Fig. 6*D*). In THP-1 cells ectopically expressing OPG188, pretreatment with NAD^+^ or TF2B similarly alleviated the inhibitory effects on the transcription of *IFNB1*, *ISG54*, and *ISG56*, as well as on the phosphorylation of STING, TBK1, and IRF3 following HSV-1 infection (Fig. 6 *E* and *F*). Although TF2B exhibited a strong rescue effect on *IFNB1* mRNA expression, it only marginally restored the expression of the downstream genes *ISG54* and *ISG56* (Fig. 6 *C* and *E*). To determine whether TF2B inhibits the downstream IFN-I signaling pathway, we performed qPCR analyses, which indicated that TF2B does not suppress IFNβ-induced transcription of *ISG15*, *ISG54*, or *ISG56* (*SI Appendix*, Fig. S5). TF2B, a monomeric tea polyphenol, is recognized as a key biologically active component in black tea (28) (Fig. 6*G*). NAD^+^ is a coenzyme essential for hydride transfer (29) (Fig. 6*H*). Molecular docking predictions indicate that both TF2B and NAD^+^ are positioned to bind within a pocket located at the dimer interface of Poxin (Fig. 6 *I* and *J*). This computationally modeled interaction within the catalytic site provides a potential structural basis for their ability to counteract OPG188-mediated suppression of the cGAS–STING signaling pathway.

### NAD+ and TF2B Potentiate cGAS–STING Pathway Induced By MPXV Infection.

**Fig. 7.**
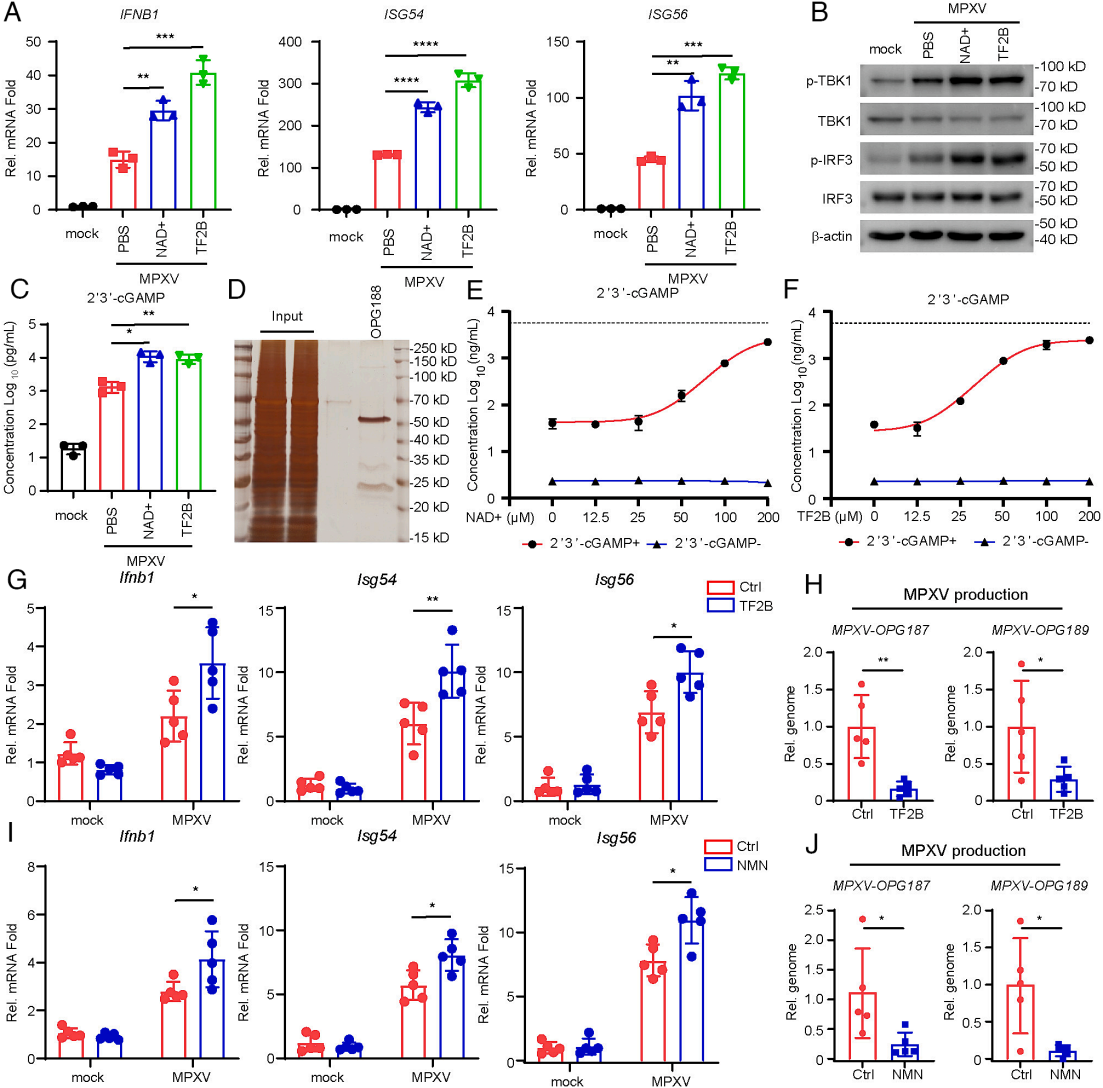
NAD+ and TF2B potentiate cGAS–STING pathway induced by MPXV infection. (*A*–*C*) THP-1 cells were treated with NAD^+^ and TF2B (final concentration: 100 μM), then infected with MPXV (MOI = 0.1) or left uninfected for 24 h before qPCR (*A*), immunoblotting (*B*), and ELISA (*C*) analysis. (*C*) Analysis of silver-stained SDS-PAGE gels for OPG188 before and after purification. (*E* and *F*) Following a 30 min incubation of 2′3′-cGAMP with purified OPG188 in the presence of NAD^+^/TF2B at 37 °C, the remaining undegraded 2′3′-cGAMP was quantified by ELISA. The dashed line represents the measured level of 2′3′-cGAMP in the baseline control group without OPG188. (*G* and *H*) Quantification by qPCR of the following in mouse lungs (n = 5 in each group): (*G* and *I*) mRNA levels of *Ifnb1*, *Isg54*, and *Isg56*; (*H* and *J*) viral load.

To further investigate the roles of NAD^+^ and TF2B, we analyzed their effects during MPXV infection. Results showed that both NAD^+^ and TF2B enhanced MPXV-induced transcription of *IFNB1*, *ISG54*, and *ISG56*, promoted phosphorylation of TBK1 and IRF3, and increased intracellular 2′3′-cGAMP levels following infection (Fig. 7 *A*–*C*). We then purified the OPG188 protein (Fig. 7*D*) and found that both compounds effectively inhibited OPG188 activity at concentrations above 100 µM (Fig. 7 *E* and *F*). In mouse studies, we used β-Nicotinamide mononucleotide (NMN), a precursor that elevates NAD^+^ levels in vivo (30, 31). Treatment with NAD^+^ (via NMN) or TF2B enhanced MPXV-induced expression of *Ifnb1*, *Isg54*, and *Isg56* after 3 days postinfection and suppressed MPXV replication after 5 days postinfection (Fig. 7 *G*–*J*).

## Discussion

The DNA sensor cGAS plays a pivotal role in host antiviral immunity. Over the past decade, numerous mechanisms by which viruses evade immune detection through antagonism of the cGAS–STING signaling pathway have been elucidated (18, 32–34). Despite encoding approximately 200 viral proteins, MPXV remains poorly characterized with respect to its inhibition of STING-mediated antiviral signaling (13, 19). A recent study reported that vaccinia virus (VACV), which belongs to the same *Orthopoxvirus* genus as MPXV, interacts with cGAS via its E5 protein, promoting cGAS ubiquitination and proteasome-dependent degradation, thereby suppressing the cGAS–STING pathway (35). However, MPXV does not encode a homolog of VACV E5, suggesting that different *Orthopoxviruses* may employ distinct strategies to inhibit STING signaling. In this study, we performed a comprehensive screen of all 179 genes encoded by the MPXV genome and identified three genes-*OPG147*, *OPG188*, and *OPG200*-that function as antagonists of the cGAS–STING signaling pathway.

A recent study identified OPG147 as a conserved inhibitor of the cGAS–STING pathway through its targeting of STING (19). In the same study, OPG188 was also found to markedly inhibit cGAS–STING-mediated activation of the ISRE in HEK293 cells, exhibiting a stronger inhibitory effect than OPG147. However, conflicting results from another study showed that OPG188 does not suppress cGAS–STING-induced IFN-I production (13). Further complexity arises from nuclease activity assays performed on 29 expressed Poxin proteins—including representatives from mammalian and insect poxviruses, parasitoid wasps, alphabaculoviruses, lepidopterans, cypoviruses, betabaculoviruses, and betaentomopoxviruses—which revealed that nine of these Poxin-containing proteins lost the ability to degrade 2′3′-cGAMP (11). Furthermore, one previous study has revealed that diverse cGAS-like receptors recognize dsDNA and dsRNA ligands and catalyze the synthesis of isomer-specific nucleotide signaling molecules, including cGAMP, c-UMP-AMP, and c-di-AMP (36). It may be that some Poxin proteins have alternate specificities for second messenger molecules and would not be expected to act on 2′3′-cGAMP. In our study, we demonstrate that MPXV-OPG188 functions as an endonuclease that degrades 2′3′-cGAMP in cells, thereby antagonizing cGAS–STING-dependent IFN-I production. Moreover, we identify key amino acid residues essential for this enzymatic activity (Fig. 5).

Previous studies have well established that residues H17, R60, Y138, K142, R182, and R184 in vaccinia virus (VACV) poxin are essential for the degradation of 2′3′-cGAMP (9). In contrast, our findings reveal that only H15, I79, K140, and R182 of MPXV-OPG188 are critical for its antagonism of cGAS–STING-mediated activation of the *IFNB* promoter in HEK293T cells (Fig. 5*F*). Among these, H15, K140, and R182 are situated within the catalytic pocket of OPG188 and display functional conservation with the corresponding residues H17, K142, and R184 in VACV. Other residues of OPG188, however, are not required for its inhibition of cGAS–STING-dependent IFN-I production. Moreover, we identified I79-a residue outside the catalytic pocket—as playing a key role in OPG188’s ability to suppress cGAS–STING signaling. This residue is critical for maintaining the structural integrity of OPG188.

In addition to OPG147 and OPG188, we identified OPG200 as a potent antagonist of the cGAS–STING signaling. OPG200 encodes a Bcl-2-like protein; previous studies have established that most viral Bcl-2 homologs encoded by poxviruses possess immunomodulatory properties mediated through diverse mechanisms (37–40). Although MPXV-OPG200 also encodes a Bcl-2-like protein, its function remains uncharacterized. In contrast, studies of its VACV homolog B13R have mainly focused on its antiapoptotic activity during viral infection (41–43). Therefore, the potential role of MPXV-OPG200 and its orthologs in other poxviruses in antagonizing the cGAS–STING pathway, along with the underlying mechanisms, remains an important question for future investigation.

NAD^+^ is an essential endogenous metabolite and coenzyme in all human cells in which it is pivotal for multiple processes including DNA repair and mitophagy. Previous studies have shown that supplementation with NAD^+^ or its precursor NMN exerts beneficial effects in aging, inflammation, and cancer therapy (31, 44). Here, we demonstrate that NAD^+^ also exhibits a moderate inhibitory effect on 2′3′-cGAMP degradation and confers limited antiviral activity. Additionally, TF2B, a key bioactive component of black tea, similarly contributes to moderate antiviral protection. Although both NAD^+^ and TF2B only partially inhibit OPG188 enzymatic activity, structural modifications based on these compounds could facilitate the development of more potent anti-mpox therapeutics.

In summary, by systematically screening the genes of MPXV, we have identified three viral genes—*OPG147*, *OPG188*, and *OPG200*—that antagonize the cGAS–STING signaling pathway. Notably, OPG188 functions as a nuclease that degrades the second messenger 2′3′-cGAMP. The structural features of the catalytic active site of OPG188 provide a basis for rational drug design or virtual screening aimed at identifying small-molecule inhibitors of its enzymatic activity. Such inhibitors (NAD^+^, TF2B) could potentiate the cGAS–STING-mediated antiviral innate immune response induced by MPXV infection (*SI Appendix*, Fig. S6). These findings enhance our understanding of both the regulation of the cGAS–STING axis and MPXV immune evasion strategies, thereby offering valuable insights for the development of therapeutic approaches against MPXV infection.

## Supplementary Material

Appendix 01 (PDF)

## Data Availability

All study data are included in the article and/or *SI Appendix*.
