# Supplementary material for: Monkeypox virus protein OPG188 antagonizes cGAS–STING antiviral signaling pathway to mediate immune evasion
Source: Proc Natl Acad Sci U S A. 2026 Mar 18;123(12):e2523334123. doi: 10.1073/pnas.2523334123 (PMC13012132; doi:10.1073/pnas.2523334123)
Supplement: Supplementary file 1 — Appendix 01 (PDF) [file pnas.2523334123.sapp.pdf]

Table S1 Conservative region mutation sites in OPG188 (C1M-C9M)

Genes	Mutation Sites
OPG188-C1M	F2A, H5A, N13A, L14A, H15A, F17A, P18A, S21A
OPG188-C2M	D27A, V28A, K30A, V36A, Y41A
OPG188-C3M	M49A, W50A, S53A, R58A, Y59A, I60A, G61A, L63A, L64A, P65A, F67A, E68A, C69A
OPG188-C4M	L73A, G76A, P78A, I79A, D81A, G84A, I87A, S88A, T91A
OPG188-C5M	N96A, Y98A, S101A, G102A, I103A, G104A, Y105A, E106A, L108A, D109A
OPG188-C6M	I117A, H119A, H120A, L122A, G125A, V128A, Y129A, G130A
OPG188-C7M	G130A, Q133A, Y136A, I139A, K140A
OPG188-C8M	G152A, I154A, D156A, Y157A, H158A, V159A, W160A, I161A, G162A, D163A
OPG188-C9M	T169A, G175A, K176A, E177A, I178A, R180A, M181A, R182A, K184A, G186A, L189A

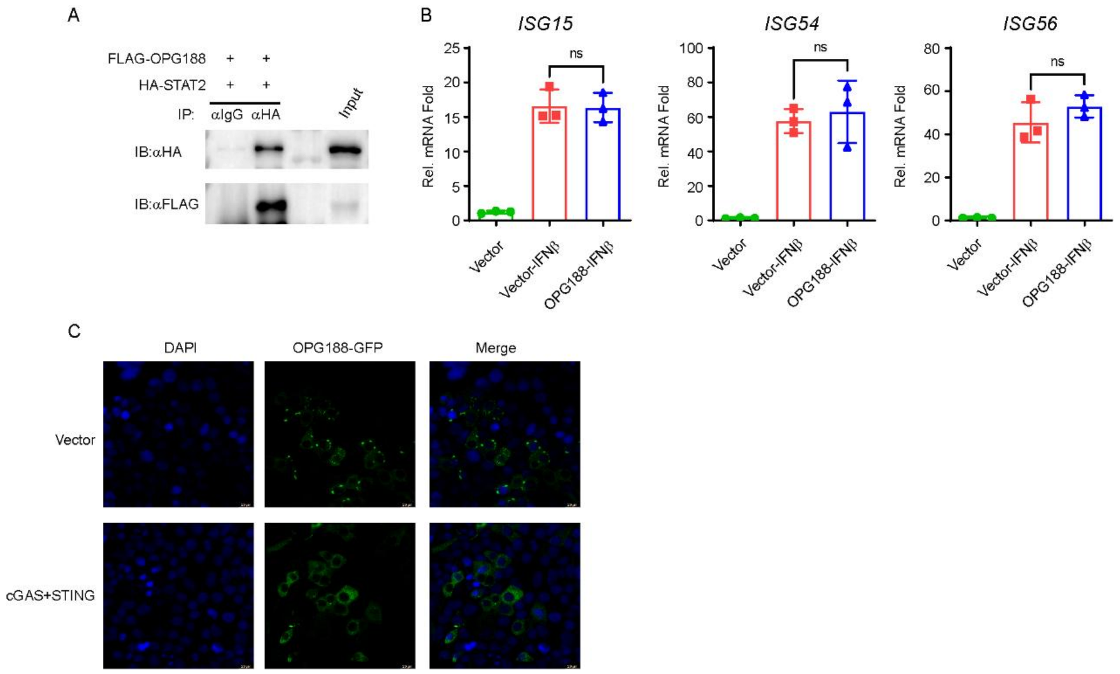

SFigure 1. OPG188 Interacts with STAT2 but Does Not Suppress IFNβ-Induced Downstream Gene Expression

(A) Association of OPG188 with STAT2. HEK293 cells (5×10^6) were transfected with the indicated plasmids. At 24 h post-transfection, co-immunoprecipitation was performed using control mouse IgG or anti-HA antibody. Immunoprecipitates and whole-cell lysates were analyzed by immunoblotting with antibodies as indicated. (B) Effect of OPG188 on IFNβ-induced gene expression. HEK293 cells were transfected with OPG188 or empty vector for 12 h, followed by treatment with or without 1000 U/mL IFNβ for an additional 12 h. Gene expression was assessed by quantitative PCR. (C) Subcellular localization of OPG188 and cGAS-STING pathway components. HEK293 cells were transfected with OPG188, cGAS plus STING, or vector control for 24 h and then analyzed by confocal microscopy.

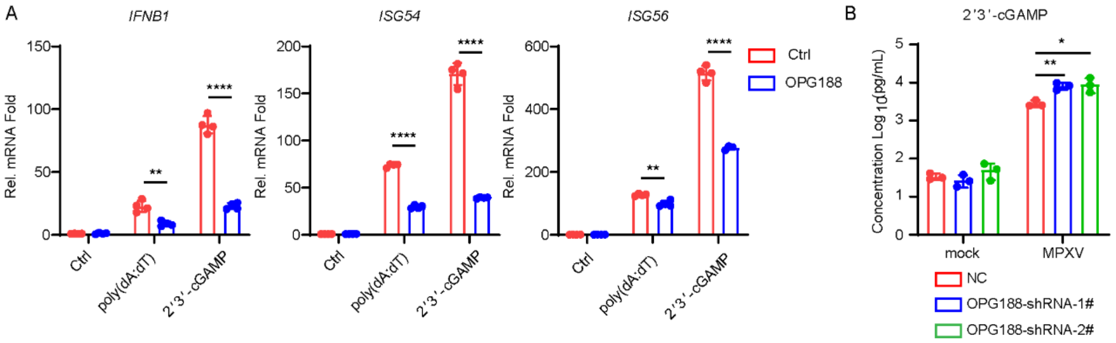

SFigure 2. OPG188 antagonizes cGAS-STING pathway by targeting 2′3′-cGAMP
(A) OPG188 suppress *IFNB1*, *ISG54*, *ISG56* expression triggered by poly(dA:dT) or 2′3′-cGAMP. THP-1 cells stably expressing OPG188-Flag or an empty vector were treated with poly(dA:dT) (1 µg/10^6 cells) or 2′3′-cGAMP (2µg/10^6 cells) for 12 h and analyzed by qPCR. (B) Knockdown of OPG188 expression enhanced intracellular 2′3′-cGAMP levels. THP-1 cells stably expressing either the indicated OPG188-shRNA or a control shRNA were infected with MPXV (MOI = 1) for 18 h and then analyzed by ELISA.

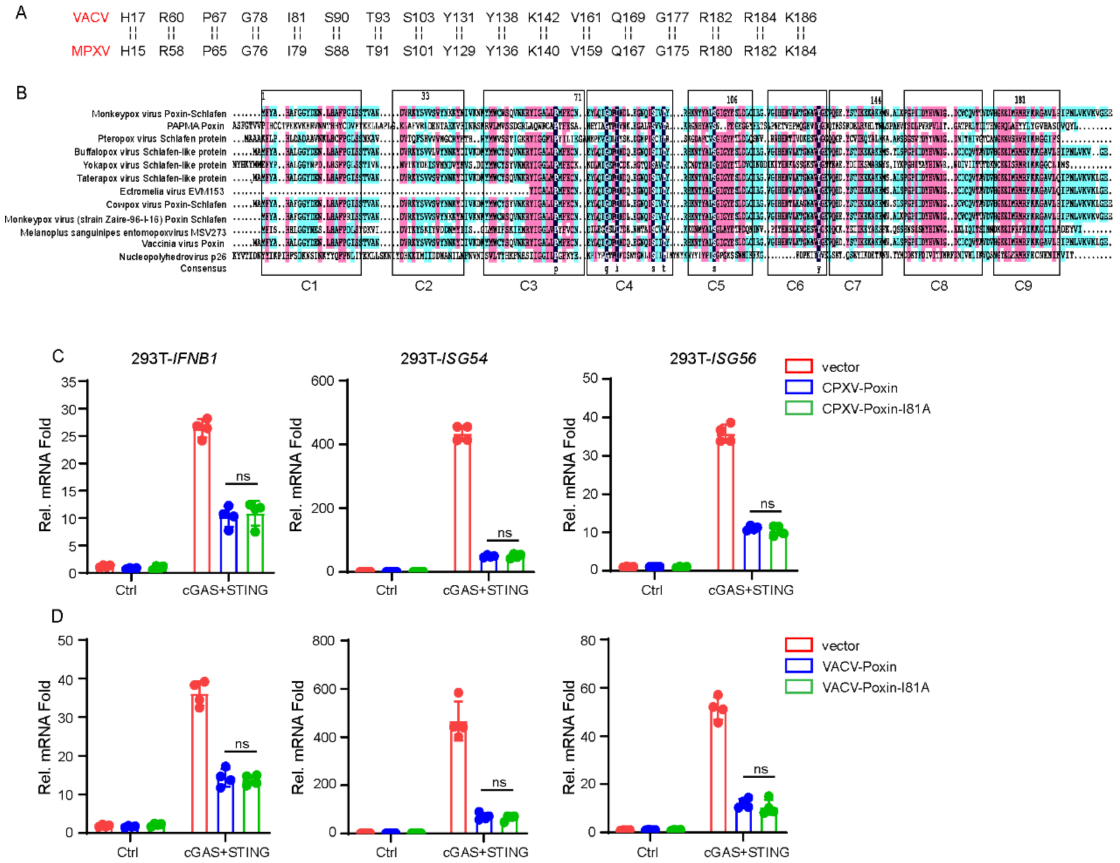

SFigure 3. Poxin-I81 on VACV and CPXV are not for antagonizing cGAS-STING signaling

(A) Amino Acid Sequence Homology Comparison of Poxin between VACV and MPXV. (B) Sequence alignment demonstrating strong conservation of Poxins across different species. (C) The I81 residue of CPXV Poxin is dispensable for antagonism of cGAS-STING signaling. HEK293T cells were transfected with plasmids encoding CPXV Poxin-WT, CPXV Poxin-I81, cGAS plus STING, or empty vector for 24 h, followed by qPCR analysis. (D) The I81 residue of VACV Poxin is not required for inhibition of cGAS-STING signaling. HEK293T cells were transfected with plasmids encoding VACV Poxin-WT, VACV Poxin-I81, cGAS plus STING, or empty vector for 24 h prior to qPCR analysis.

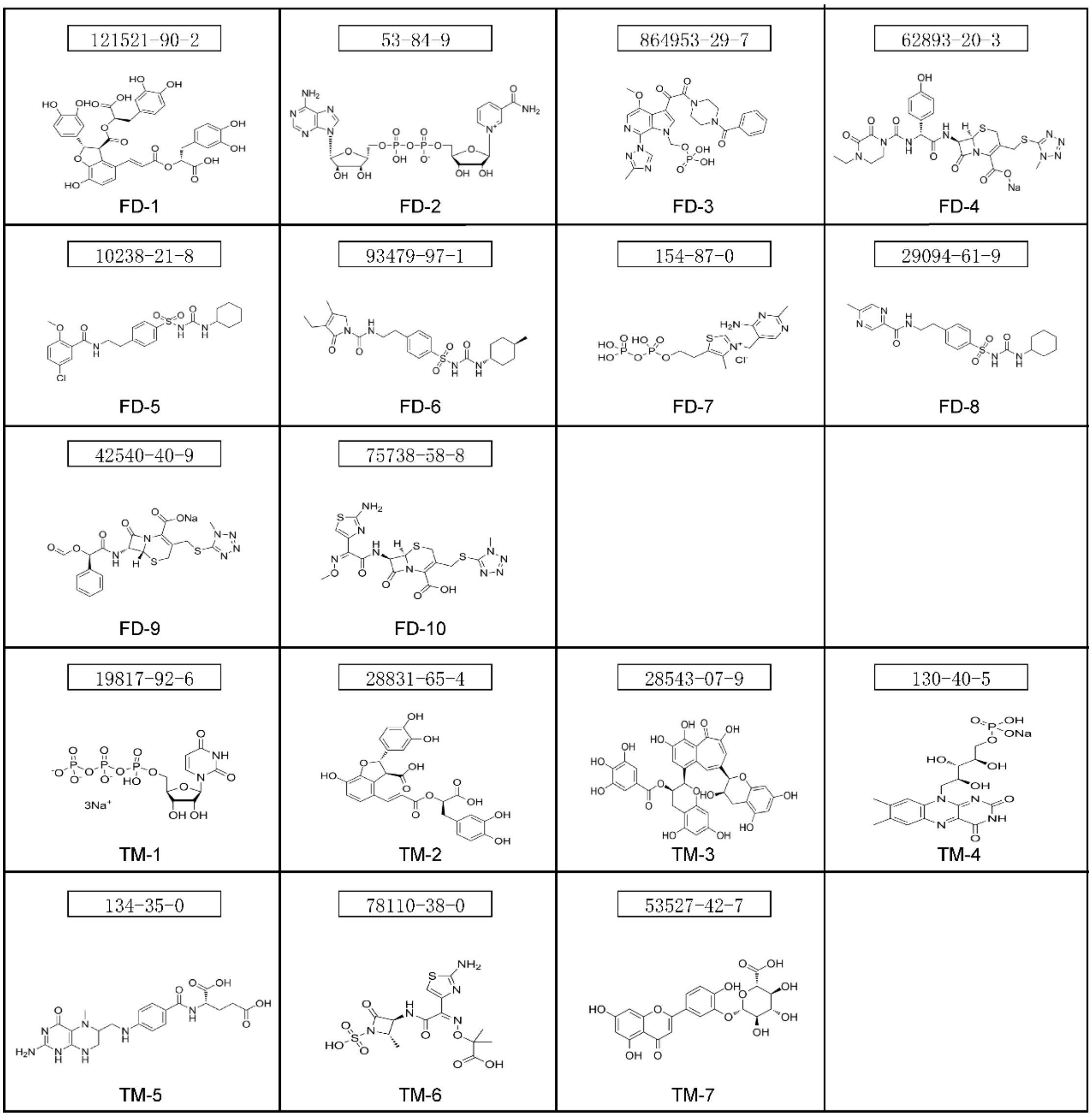

SFigure 4. Top 17 candidate compounds identified through molecular docking and virtual screening.

Compounds are sourced from the FDA (FD) and TargetMol (TM) libraries. Each compound is annotated with its CAS registry number (top), chemical structure (middle), and compound identifier used in this study (bottom).

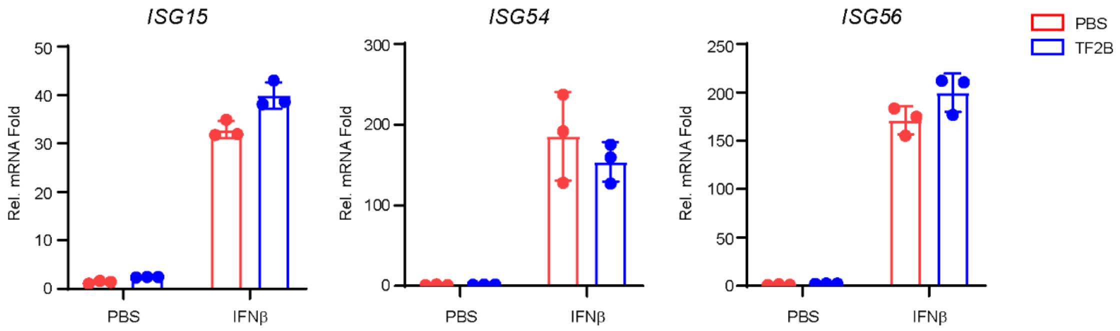

SFigure 5. TF2B not inhibit IFNβ downstream genes transcription

HEK293T cells (1×10^5) were transfected with IFN-β reporter (50 ng), pRL-TK reporter (5 ng). Addition of TF2B (final concentration: 100 μM) and IFNβ (1000 U/mL) at 12 h post-transfection followed by 12-hour incubation prior to qPCR assay.

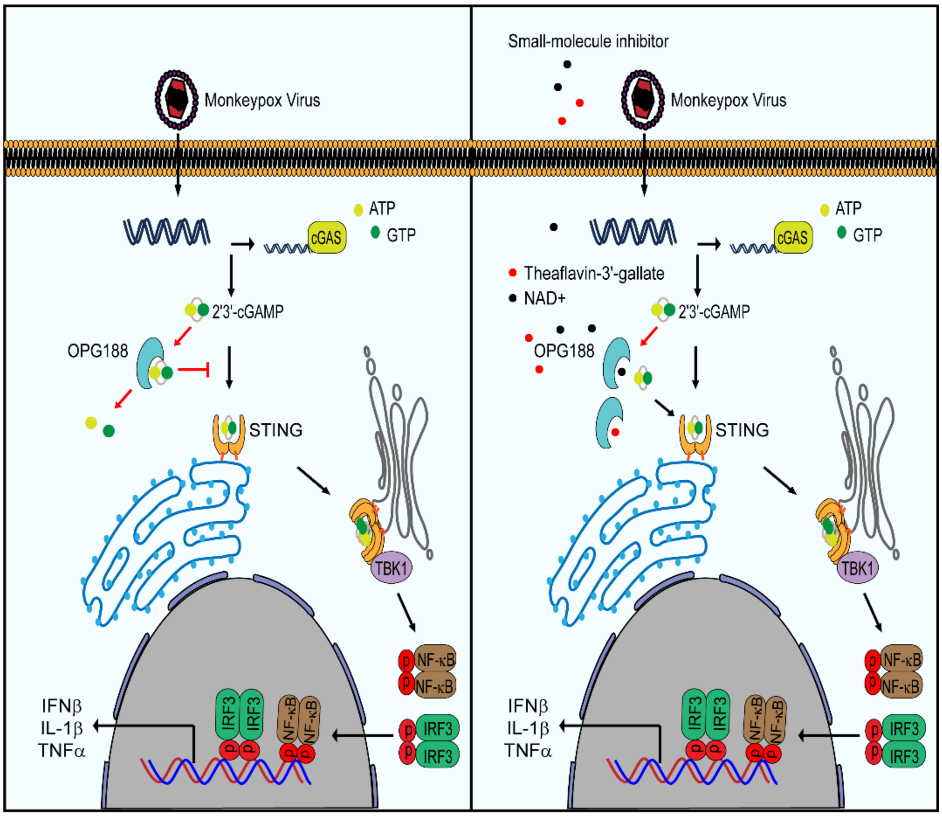

SFigure 6. Working model of OPG188 antagonizes cGAS-STING antiviral signaling pathway and TF2B/NAD^{+} antagonizes OPG188-mediated regulation of the cGAS-STING signaling pathway

Materials and Methods

Cells and viruses

HEK293T, THP-1 and Vero cells were obtained from the American Type Culture Collection. HEK293T cells and Vero cells were grown in DMEM supplemented with 10% FBS in 5% CO2. THP-1 cells were grown in RPMI-1640 supplemented with 10% FBS in 5% CO2. MPXV (Clade IIb) was provided by Dr. Jin Changzhong (The First Affiliated Hospital, Zhejiang University School of Medicine). MPXV-ZJU-01/2023 was isolated in 2023 from a mpox patient in Hangzhou. MPXV whole Genome Shotgun project has been deposited at GENBASE under the accession C_AA040049. MPXV stock was prepared on Vero cells. HSV-1 and SeV were stored in our own laboratory. HSV-1 stock was prepared on Vero cells and SeV stock was prepared on chicken embryo.

Reagents and antibodies

Plasmid Construction: ClonExpress II One Step Cloning Kit (Vazyme, C112); Mut Express Universal Fast Mutagenesis Kit (Vazyme, C216); PrimeSTAR® HS (TaKaRa, R040A). Antibodies against FLAG-Tag (ABclonal, AE005); HA-Tag (ABclonal, AE105); Phospho-STING (ABclonal, AP1369) STING (ABclonal, A21051); Phospho-TBK1 (CST, 5483S) TBK1 (Diagbio, db11086); Phospho-IRF3 (Abways, CY6575); IRF3 (ABclonal, A19717); Beta-Actin antibody (ABclonal, AC026). Calcium phosphate precipitation (Beyotime, Catalog No. C0508), *Evo M-MLV* RT Kit with gDNA clean for qPCR (Accurate Biology, AG11711), TRIzol reagent (Invitrogen™, 15596026CN), SYBR Green Mix (Accurate Biology, AG11736); Luciferase assay kit (Vazyme, DL101), M-PER lysis buffer (ThermoFisher, 78501), 1× EDTA-free protease inhibitor cocktail (Roche, 4693132001), 2'3'-cGAMP ELISA Kit (Cayman, 501700), HisPur™ Ni-NTA (Thermo, 90099), Silver staining kit (Beyotime, P0017S), β-Nicotinamide mononucleotide (NMN) (MCE, 1094-61-7), Theaflavin-3'-gallate (TF2B)(MCE, 28543-07-9), NAD+ (MCE, 53-84-9), 2'3'-cGAMP (MCE, 1441190-66-4), Protein G magnetic bead (Beyotime, P2105).

Plasmid construction details and sources

The expression clones encoding 179 independent MPXV-encoded proteins were constructed by standard molecular biology technique. In brief, the gene encoding the MPXV protein was obtained by DNA fragment synthesis or PCR amplification and subsequently cloned into the pcDNA™3.1/myc-His vector (Invitrogen, V855-20). The construction of point mutation overexpression plasmids was carried out following the detailed protocol provided in the Mut Express Universal Fast Mutagenesis Kit manual. Constructed plasmids were verified by DNA sequencing. Expression plasmids for FLAG- or GFP-tagged OPG188 and its mutants; mCherry-tagged GalT (Golgi marker), HA-tagged RPS18, OPG188-shRNA plasmid, HA-tagged STAT2 were constructed by

standard molecular biology techniques. cGAS, STING, TBK1, IRF3-5D, IFNβ/ISRE/NF-κB luciferase reporter plasmids were kindly provided by Dr. Pan Chaohu (The Third People's Hospital of Shenzhen). HA-tagged STAT2 were kindly provided by Nie Ying (Guizhou Medical University).

Buffers utilized throughout the experimental process.

SDS lysis buffer (50 mM Tris-HCl pH 6.8, 4% SDS, 0.01% bromophenol blue, 20% glycerol, 100 mM DTT); Lysis buffer used in the SDD-AGE assay (0.5% Triton X-100, 50mM Tris-HCl, 150mM NaCl, 10% Glycerol); NP-40 lysis buffer (20 mM Tris-HCl [pH 7.4], 150 mM NaCl, 1 mM EDTA, 1% Nonidet P-40, 10 mg/ml aprotinin, 10 mg/ml leupeptin and 1 mM phenylmethylsulfonyl fluoride); Reaction buffer for OPG188 nuclease activity assays (50 mM HEPES-KOH (pH 7.5), 35 mM KCl, and 1 mM DTT).

Constructs of stable cell lines

HEK293T cells (5×10^6) were transfected with the indicated retroviral plasmids, along with pRRE (4.1 μg), pREV (2 μg), pVSV-G (3 μg) (OPG188 and its mutants overexpression plasmids (4 μg)) or pMD2.G (4 μg), psPAX2 (3 μg) (OPG188 knockdown plasmids (1 μg)) packaging plasmids by calcium phosphate precipitation. 12 hours post-transfection, the culture medium was replaced with fresh antibiotic-free medium. After an additional 48 hours, the supernatant of virus-containing cell culture medium was collected, filtered through a 0.45 μm membrane (Millipore, SLHV033RB), and used to infect THP-1 cells for 4 days. The infected THP-1 cells were selected by flow cytometry before experiments.

Transfection and reporter assays

HEK293T cells (2×10^4) were transfected using calcium phosphate precipitation in 96-well cell culture plate. Control plasmids were added to ensure that each transfection receives the same amount of total DNA. In reporter assays, pRL-TK (Renilla luciferase, 5 ng) reporter plasmid was transfected as an internal control. Luciferase activity was measured using luciferase assay kit and detected by GloMax® 96 Microplate Luminometer (Promega).

2′3′-cGAMP ELISA

Figure 7C and SFigure 2B: Around 1×10^6 THP-1 cells were lysed in 0.4 mL M-PER lysis buffer in the presence of 1× EDTA-free protease inhibitor cocktail. Cells were lysed at 4°C for 10 min with agitation. Insoluble cell debris was pelleted by centrifugation at 15000×g for 10 min. The supernatant was then used for quantification of 2′3′-cGAMP content with the 2′3′-cGAMP competition ELISA kit.

Figure 7E and 7F: After a 30 min incubation at 37 °C, the OPG188 reaction mixture was diluted 1000-fold in PBS, and the remaining undegraded 2′3′-cGAMP in the mixture was quantified using the ELISA kit.

Detection of 2′3′-cGAMP by Mass Spectrometry

HEK293T cells were transfected with either the OPG188 plasmid or an empty vector

control. After 24 hours, cells (1×10^6) were harvested, washed three times with PBS, and lysed using 1 mL of NP-40 lysis buffer. The lysate was centrifuged at 12,000 × g for 2 minutes, and 100 μL of the supernatant was aliquoted into a PCR tube. Then, 1 μL of 2′3′-cGAMP (1 mg/mL) was added to the supernatant, and the mixture was incubated at 37 °C for 4 hours. Prior to mass spectrometric analysis, samples were diluted 50-fold with ddH_2O to quantify changes in 2′3′-cGAMP levels within the reaction system.

Gene-specific primer sequences for qPCR

18S: CCGGTACAGTGAAACTGCGAATG (forward) and
GTTATCCAAGTAGGAGAGGAGCGAG (reverse),
IFNB1: GACAGGATGAACTTTGACATCCC (forward) and
CTCAACAATAGTCTCATTCCAGCC (reverse),
Ifnb1: GCCTTTGCCATCCAAGAGATGC (forward) and
ACACTGTCTGCTGGTGGAGTTC (reverse),
ISG15: CTCTGAGCATCCTGGTGAGGAA (forward) and
AAGGTCAGCCAGAACAGGTCGT (reverse),
ISG54: GGAGCAGATTCTGAGGCTTTGC (forward) and
GGATGAGGCTTCCAGACTCCAA (reverse),
ISG56: GCCTTGCTGAAGTGTGGAGGAA (forward) and
ATCCAGGCGATAGGCAGAGATC (reverse),
Isg54: CGAACTACCGTCTGGATGACTG (forward) and
CTTCAACCAGCGCCATTGCTTG (reverse),
Isg56: TACAGGCTGGAGTGTGCTGAGA (forward) and
CTCCACTTTCAGAGCCTTCGCA (reverse),
OPG187: TAGATGCGGTGATCAGAGCCAA (forward) and
CTCCGTGAGAATATCCTTGCTCG (reverse),
OPG188: TTTTACGCACACGCTTTCGGTG (forward) and
TTACACCACATATATTTGTTTT (reverse),
OPG189: GATGCCGAAGACAGTTACGGTT (forward) and
CGCGTTGTCGATATCGTGTT (reverse),

Coimmunoprecipitation

HEK293T cells (~10^7) were lysed with 1 ml NP-40 lysis buffer for 30 minutes on ice. Cell lysates were clarified by centrifugation at 12,000 rpm for 10 minutes (4°C). For each immunoprecipitation, the cell lysate (0.8 mL) was incubated with the indicated antibodies (0.5 μg) and protein G magnetic bead (25 μL) at 4°C for 4 hours. The protein-bound beads were then collected and washed three times with NP-40 lysis buffer (0.75 mL) containing 0.5 M NaCl. Subsequently, the beads were then resuspended in 50 μL of SDS lysis buffer and lysed by incubation at 97°C for 15 min, followed by immunoblot analysis.

Confocal microscopy

HEK293T cells were transfected with either OPG188 or an empty vector, in conjunction with cGAS, STING, and mCherry-GalT, for 24 hours. Following transfection, the cells were fixed with 4% paraformaldehyde at 37 °C for 15 min and washed three times with PBS. Cells were then permeabilized using 0.5% Triton X-100 for 20 min at room temperature and washed three times with PBS for 5 min each. After blocking with 5% BSA for 1 h, cells were incubated overnight with an anti-STING primary antibody. The primary antibody was removed, and the cells were washed three times with PBS (5 min per wash). A secondary antibody was applied and incubated for 2 h at room temperature in the dark. Following three additional PBS washes, nuclei were stained with DAPI (Beyotime, C1006). Finally, the cells were imaged using Leica STELLARIS 5 confocal microscope under a 100× oil objective.

OPG188 Protein Purification

HEK293T cells (around 1×10^6 cells per dish) cultured in 10-cm dishes were transfected with 10 μg of pCDNA3.1-OPG188-6×His plasmid. After 36 h, cells from ten 10 cm dishes were harvested and lysed using NP-40 cell lysis buffer supplemented with protease inhibitor cocktail. The lysates were centrifuged at 15,000 × g for 10 min, and the resulting supernatants were subjected to purification of OPG188 protein using HisPur™ Ni-NTA. Purified OPG188 was concentrated by Amicon® Ultra-10 kDa (Millipore, UFC801008) and washed five times with PBS. A small aliquot of the concentrated OPG188 protein was analyzed by SDS-PAGE and visualized by silver staining kit. The remaining protein was supplemented with 10% glycerol and stored at -20°C for future use.

Docking and Virtual Screening

All works of virtual screening (VS) were conducted in Yinfo Cloud Platform (https://cloud.yinfotek.com/). The prepared FDA and TargetMol libraries, containing 2,858 and 4,279 compounds respectively, were employed for VS. The crystal structure of MpxV protein (PDB code: 8P44) was automatically downloaded from the RCSB Protein Data Bank (http://www.rcsb.org/). All redundant atoms except chain A and B were deleted and then the protein structure carefully treated in several steps including residue repairing, protonation, and partial charges assignment in AMBER ff14SB force field. The DMS tool was employed to build molecular surface of the receptor using a probe atom with a 1.4 Å radius. The binding pocket was defined by the crystal ligand (WY8) and spheres were generated filling the site by employing the Sphgen module in UCSF Chimera(1). A box enclosing the spheres was set with center of (23.328, 44.353, 63.707) and sizes of (27.964, 26.556, 28.556). DOCK 6.9 (2, 3) program was utilized to execute semi-flexible docking where 10000 different orientations were produced. Clustering analysis was performed (RMSD threshold was set 2.0 Å) for candidate poses and the best scored one was output for each compound. Those compounds with Grid score larger than -70 kcal/mol were filtered. Finally, potential hits were identified

through manual analysis and selection, and binding modes were analyzed by PyMOL (http://pymol.org/citing).

NMN or TF2B against OPG188 nuclease activity assays in vitro

Purified OPG188 (200 ng) was incubated with varying concentrations of NAD+/TF2B for 10 minutes at 37 °C in a 50 μL reaction buffer containing 50 mM HEPES-KOH (pH 7.5), 35 mM KCl, and 1 mM DTT. Subsequently, 2′3′-cGAMP (10 μM) was added to the reaction mixture and incubated for an additional 30 minutes at 37 °C. Control reactions were carried out under identical conditions without the addition of 2′3′-cGAMP. Additionally, 2′3′-cGAMP (10 μM) in reaction buffer alone was processed under the same conditions as a baseline control. After incubation, each reaction mixture was diluted 1000-fold with PBS, and the concentration of 2′3′-cGAMP was measured by ELISA using a standard curve for quantification.

MPXV infection of mice

All animal experiments were conducted using BALB/c mice (female, 6 weeks) housed in an Animal Biosafety Level 3 facility. One week prior to infection, mice received intraperitoneal injections of either the NAD+ precursor nicotinamide mononucleotide (NMN; 500 mg/kg) or vehicle control (PBS) for four consecutive days. The dose of NMN were previously described(4). In parallel, TF2B (10 mg/kg/day) or vehicle (PBS) was administered daily by oral gavage beginning one week before infection. Mice were then intranasally challenged with MPXV at a dose of 5×10^6 TCID50 per mouse. Treatment with NMN or TF2B was continued after viral infection. Lung tissues were collected at 3- and 5-days post-infection for subsequent pathological evaluation and qPCR analysis.

SI References

1. Pettersen EF, *et al.* (2004) UCSF Chimera--a visualization system for exploratory research and analysis. *J Comput Chem* 25(13):1605-1612.
2. Lang PT, *et al.* (2009) DOCK 6: combining techniques to model RNA-small molecule complexes. *RNA* 15(6):1219-1230.
3. Mukherjee S, Balius TE, & Rizzo RC (2010) Docking validation resources: protein family and ligand flexibility experiments. *J Chem Inf Model* 50(11):1986-2000.
4. Han S, *et al.* (2023) Mitochondrial integrated stress response controls lung epithelial cell fate. *Nature* 620(7975):890-897.
